# Complete Genome Sequence of the Multiresistant *Acinetobacter baumannii* Strain AbH12O-A2, Isolated during a Large Outbreak in Spain

**DOI:** 10.1128/genomeA.01182-14

**Published:** 2014-11-13

**Authors:** M. Merino, L. Alvarez-Fraga, M. J. Gómez, A. M. Aransay, J. L. Lavín, F. Chaves, G. Bou, M. Poza

**Affiliations:** a Servicio de Microbiología, Instituto de Investigación Biomédica (INIBIC), Complejo Hospitalario Universitario (CHUAC), Sergas, A Coruña, Spain; b Departamento de Microbiología Clínica, Hospital Universitario 12 de Octubre, Madrid, Spain; c Departamento de Evolución Molecular, Centro de Astrobiología INTA-CSIC, Madrid, Spain; d Plataforma de Análisis Genómico CIC bioGUNE & CIBERehd, Derio, Spain

## Abstract

We report the complete genome sequence of *Acinetobacter baumannii* strain AbH12O-A2, isolated during a large outbreak in Spain. The genome has 3,875,775 bp and 3,526 coding sequences, with 39.4% G+C content. The availability of this genome will facilitate the study of the pathogenicity of the *Acinetobacter* species.

## GENOME ANNOUNCEMENT

*Acinetobacter baumannii* is a major cause of hospital-acquired infections. Its success in the hospital environment lies in its genetic versatility and its ability to persist in extreme conditions. During 2006–2008, 377 patients were colonized/infected with multiresistant *A. baumannii* strains in a hospital in Madrid, Spain. This was one of the most important nosocomial outbreak produced by *A. baumannii* known to date (1, 2). The clones isolated during the outbreak harbored different plasmids, all containing a *bla*_OXA-24_ gene mobilized through a Xer recombination mechanism. Two mutations found to modify the gene expression of virulence factors provided an advantage to strain AbH12O-A2, whose genome sequence is described here. Previous studies have described the molecular mechanisms involved in the spread and epidemiology of this strain (1–6).

The total genomic DNA isolated from strain AbH12O-A2 was sheared into smaller fragments with the Covaris S/E210 system. Specific adapters were ligated to DNA fragments, and libraries containing inserts of 500 bp were purified and selectively enriched by PCR. After quality and quantity controls, 90 nucleotide fragments were paired-end sequenced in an HiSeq2000 system, with the TruSeq PE cluster kit version 3, cBot-HS, and TruSeq SBS kit-HS version 3 (Illumina Inc.). Once the gaps were identified, pairs of primers were designed and specific regions were amplified. Products were sequenced by BigDye Terminator version 3.1 chemistry in an ABI Seq Instrument (Applied Biosystems). After quality control of the raw data, clean data were aligned to *Acinetobacter baumannii* ATCC 17978, as a reference sequence. Assembly of the short reads into the genome sequence was performed using the SOAPdenovo version 1.05 program (http://soap.genomics.org.cn/soapdenovo.html). Key parameter K setting at 47 is determined by optimal assembly according to the paired-end and overlap relationship via mapping reads to contigs.

The *A. baumannii* AbH12O-A2 complete genome consists of 3,875,775 bp with a G+C content of 39.4% and contains 3,526 coding sequences, 72 tRNA genes, and 6 rRNA clusters. *A. baumannii* ACICU (score 518), *A. baumannii* AB900 (score 500), and *A. baumannii* AYE (score 483) were the closest neighbors to strain AbH12O-A2. Moreover, 56 genes associated with resistance to antibiotics and toxic compounds were found. The ResFinder bioinformatic application (7) was used to identify genes coding for antibiotic resistance. The following resistance genes were detected in the genome: *aac(3)-lla*, *strA*, and *strB* conferring resistance to aminoglycosides; *bla*_OXA-65_, *bla*_ADC-25_, and *bla*_TEM-1B_ conferring resistance to β-lactams; and *sul*2 conferring resistance to sulfonamides. Comparison of the AbH12O-A_2_ genome with other completed genomes of *A. baumannii* by use of Mauve version 2.3.1 (8) revealed a resistance island (3,612,846–3,637,461 bp) flanked by two sequences of IS*Aba1* in opposite directions. PHAST (9) analysis revealed four putative intact phages integrated in the genome, two of which are similar to *Acinetobacter* phage *Bphi*-B1251 and another two that have not previously been identified in *A. baumannii*: one is similar to Enterobacteria phage *mEp*235 and the other is similar to *Haemophilus* phage SuMu.

### Nucleotide sequence accession number.

This whole-genome shotgun project has been deposited in GenBank under accession number CP009534.
